# The Lineage of Children Born by Sperm Donation: A Shiite Perspective 

**DOI:** 10.22074/ijfs.2015.4557

**Published:** 2015-10-31

**Authors:** Saeid Nazari Tavakkoli

**Affiliations:** Department of Jurisprudence and Essentials of Islamic Law, Faculty of Theology and Islamic Studies, Tehran University, Tehran, Iran

**Keywords:** Kinship, Lineage, Intimacyn

## Abstract

**Background:**

Despite the meager role of the masculine agent in infertility (the low number of infertile men than women infertile), there are men whose wives are unable to become pregnant due to the absence of sperm, decreased numbers of sperm or lack of sufficient motile sperm. Utilizing donated sperm is a method that enables these families to have children. The use of this method prompts us to ask different questions, among which is the quality of the child’s relation to the sperm donor, the sterile man and his wife. In this research we intend to study the issue of lineage of those who use heterogeneous insemination.

**Materials and Methods:**

This analytical-descriptive research gathered relevant data in a Literature search. After a description of the fundamentals and definitions, juridical texts were subsequently analyzed and one of the viewpoints regarding lineage related to infertility treatment by donated sperm was selected.

**Results:**

There are three persons that have a possible legal relationship to the child born from this method -the sperm donor (biological father), the wife’s husband (social father) and the wife (mother). In treating infertility with donated sperm, there is neither a third party to make the possibility of attribution of the child nor is there a doubt that the child is the result of insemination of the woman’s egg with the donated sperm rather than the husband’s sperm as he has a lack of sperm.

**Conclusion:**

The child born by heterogeneous insemination only has a relation with the sperm donor and the woman contributing her egg. This child is eligible for all parental rights and obligations. These children are not related to the sterile man.

## Introduction

In order to comprehend fertility, it is important to understand how conception naturally occurs. First, the ovary must release ( ovulate ) an egg, which must be picked up by the fallopian tubes. Sperm must travel through the vagina into the uterus and into the fallopian tube in order to fertilize the egg. Fertilization usually takes place in the fallopian tube. Then, the fertilized egg or embryo travels down to the uterus, where it implants in the uterine lining and develops. Infertility occurs when there is a problem at any part of this process (1). 

Infertility is defined as one year of unprotected intercourse that does not result in pregnancy. This condition may be further classified as primary infertility, in which no previous pregnancies have occurred and secondary infertility where a prior pregnancy, although not necessarily a live birth, has occurred (2). 

Infertility affects approximately 10 to 15% of couples and is a medical problem for 2.7 million women of reproductive age in the United States. Over the past few decades, successful treatments for all infertility types have been developed, providing hope for infertile couples (3). 

The physician’s initial visit with the infertile couple is the most important because it can serve as a guide. Factors from either or both partners may contribute to difficulties in conception; therefore, it is important to consider all possible diagnoses before pursuing invasive treatment. The main causes of infertility include male factor, decreased ovarian function and ovulatory disorders ( ovulatory factor ), tubal injury, blockage, or paratubal adhesions ( endometriosis with evidence of tubal or peritoneal adhesions ), uterine factors, systemic conditions ( infections or chronic diseases such as autoimmune conditions or chronic renal failure ), cervical and immunological factors, and unexplained factors ( endometriosis with no evidence of tubal or peritoneal adhesions ) (4). 

Although 20% of infertility relates to men ( male factor ), it may be a contributing factor in as many as 30 to 40% of cases. Treatment of reversible endocrine or infectious causes of subfertility, such as sexually transmitted diseases and thyroid disorders, tends to be efficacious. Although the prevalence of infertility has not changed, the demand for infertility care has increased significantly over the past few decades (4). 

Heterogeneous insemination is a type of assisted reproductive technique ( ART ). In this method, sperm from a man other than the husband is inseminated together with the wife’s egg inside her womb. Although it is possible to use fresh sperm, in most cases frozen sperm stored in sperm banks are utilized. 

Regardless of whether Islamic jurisprudence considers the use of donated sperm as permissible ( javaz ) for the fertilization of a man’s wife or not ( adam al-javaz ), the most important question that confronts Muslim scholars is, as a result of this process, the child or children’s legal status and to whom their lineage ( nasab ) should be linked. 

## Materials and Methods

The study of lineage of children born by sperm donation is an interdisciplinary research that includes both medical and Islamic jurisprudence sciences. Identification and specification that pertain to methods to conceive a child by heterogeneous insemination is an issue related to medicine. However the specification of religious statements on this issue which include familial relative or non-relatives of children born by sperm donation is related to Islamic jurisprudence. Islamic jurisprudence is the result of Muslim jurisprudents’ attempts with specific religious beliefs. 

Due to the extensiveness of Islamic perspectives, we conducted this research according to the ideas of Shi’ite jurisprudents among the five Islamic schools of thought, namely Shi’a, Hanafi, Maliki, Shafi’i, and Hanbali. The author studied law books and Shi’ite jurisprudence texts that pertained to children’s lineage. After library research and analytical analyses, this study has proven the lack of existence of a familial relationship between the child born by sperm donation and his/her social father. 

## Results

If donated sperm is used for treating infertility, there will be three persons that have a possible legal relationship to the child born from this method-the sperm donor ( biological father ), the wife’s husband ( social father ) and the wife ( mother ). 

### Sperm donor (biological father)

There are three different viewpoints on the fatherhood relationship between the sperm donor and the child produced from this donation. 

### First viewpoint

#### Discontinuity of lineage (nasab)

Adherents of this viewpoint have cited two points in proving discontinuity of lineage. 

#### Absence of marriage bed (farash)

Some jurists believe that the child is not related to the sperm donor in terms of lineage ( nasab ) because this child has not been born in his farash (5). 

The "farash rule" makes sense when there is doubt in attribution of the child to the legal husband of the wife or a third party (6). However, in treating infertility with donated sperm, there is neither a third party to make the possibility of attribution of the child nor is there a doubt that the child is the result of insemination of the woman’s egg with the donated sperm rather than the husband’s sperm as he has a lack of sperm. 

### Lack of sexual intercourse (penetration)

In some juridical texts there are three conditions stated required for linking the lineage of the child to the husband of a woman: sexual intercourse ( dokhul ) of the man and woman, having past at least 6 months from the time of intercourse until the delivery date ( minimum delivery period ) and having past not more than 10 months from intercourse until the delivery date ( maximum delivery period ) (7,9). Therefore if the man did not have intercourse with his wife, the child born from that woman would not be linked to him. The husband would not be regarded as the father of this child (10,11). Additionally, because the sperm donor did not have intercourse with the infertile woman, the resultant child could not be considered his child (5). 

Answer: Although the issue of validity of sexual intercourse in different jurisprudent discussions such as the child’s lineage in temporary ( mowaqqat ) and permanent ( daim ) marriage, the necessity of observing the waiting period, oath of imprecation ( lian ) and denial of the child ( nafy al-walad ) is described. However it seems that the validity of sexual intercourse in substantiation of lineage and establishment of kinship between the biological father and the child is only because in normal conditions and in most cases, sexual intercourse is the way to transfer sperm into the woman’s womb (8,12,13). Therefore if a man transfers his sperm into his wife’s womb without having sexual intercourse, no doubt the resultant child will be considered his child (14,17). For example, as in anal intercourse ( watye fi al-dobor ), if there is a possibility for sperm to reach the womb, the child will be attributed to the owner of the sperm (10,18). This intercourse under normal conditions cannot be the cause for fertility (9,19). Also if the husband has been away from his wife ( ghaybat ) for more than ten months or the couple are united in the absence of intercourse, the child would not be attributed to the husband since during this period intercourse was not possible (20,22) without a need for the husband to deny the attribution of the child to himself by liʿan va nafy al-walad (23). 

### Second viewpoint

#### Difference between cases (separation)

Followers of this doctrine contend that although there is a birth relationship between the child and sperm donor, this relationship legally does not always have equal consequences. A distinction should be made between the cases. 

### Known or unknown sperm donor

In heterogeneous insemination from a known donor, the child is attributed to the donor; the parental rights and duties are preserved between them. However if the sperm donor is unknown, since the basis of donating sperm is on not having a link with the resultant child, a legal judgment on realizing the legal effects of the paternal relationship between the donor and child will be an imposition of lineage on the sperm donor. Hence the social father is the real father of the newborn child (24). 

Answer: There is no doubt that the sperm donor, whether known or unknown, has no motive for reproduction and consequently survival of generations. Thus the issue of imposition of lineage is not applied just to the unknown sperm donor. Furthermore, lineage ( nasab ) is a genetic link independent of the will of either the man or woman. For the same reason, children born without the will of either the wife, husband or both parents ( unwanted child ), have as much lineage link to their parents as the children who are born willingly (25,26). 

Unknown status does not negate lineage from the sperm donor but only prevents actualizing lineage to him. For this reason if the sperm donor is known, no doubt, the child will be attributed to him since a child without a father does not have any position in the legal system. 

### Awareness and unawareness of the sperm donor

Certain lawyers believe that awareness and unawareness of the sperm donor will affect the attribution and lack of attribution of the child to the donor. If the sperm donor, knowing that his donation will set the stage for producing a child unlawfully, proceeds to do such an action then the child born by sperm donation will be regarded as illegitimate ( walad al-zina ) and no legal relationship will exist between him and the donor. A child belongs to the father when it is born through legal and normal ways ( marriage ). Pregnancy through sperm donation is not considered to be included in such methods. 

However if the sperm donor is unaware that donating his sperm is the beginning for producing a human being or is not aware that donating sperm to others is not permissible, the child born in this way will be considered a child of suspicious intercourse ( walad al-shobha ) and therefore attributed to the sperm donor (27,28). 

Answer: The title illegitimate child ( walad alzina ) applies to a child who is the product of illegal sexual intercourse ( zina ). On the other hand, procreation is a natural process which depends upon the man’s sperm and woman’s egg to join together normally through sexual intercourse. Naturally this genetic link cannot be legally taken away (29) although legislators can ignore certain consequences of such a genetic link. 

Therefore there is no doubt that the sperm of an adulterer is not respectable ( without legal value and consequences ) (30) and the child who is born through an illegal relationship ( adultery ) is not a legitimate child (31). However this does not mean that the child born through an illegal relationship is without parents (32) because the performance of sexual intercourse itself is enough for this link (23). Although the legislator of Islam has excluded only the child’s inheritance from parents (33,35), they have recognized other outcomes of this birth such as being intimate ( mahram ) and sanctity of marriage, as is the case for legitimate children (17,29). 

Hence, the child born by heterogeneous insemination is not illegal, and there is no reason for negation of their lineage ( nasab ) and the sperm donor ( biological father ) would be their real father. 

### Third viewpoint

#### Continuation of lineage

The child born through artificial insemination with donated sperm ( AID ) legally belongs to the sperm donor attributed to him by name, biologically, genetically and customarily (24). Similarly, the sperm donor is considered to be the child’s father and the child is deemed as his real and legal child. Consequently, overall parental rights and obligations apply to the sperm donor and the child. 

To prove this claim, we cite a number of traditions ( rawayat ) as evidence such as the transfer of sperm by a method other than conventional sexual intercourse ( tribadism/lesbian sex-musahiqa ). These traditions emphasize that when a man has a type of sexual relationship with his wife that leads to ejaculation of sperm and the woman immediately leaves her husband and goes to another woman where contact between their genitals ( musahiqa ) causes the transfer of her husband’s sperm to the second woman, which results in pregnancy of the second woman by this contact. The resultant child belongs to the husband of the first woman, as the owner of sperm, in terms of lineage (36,39). 

Shiite jurists and lawyers based on these traditions have declared that once the sperm of a man enters the womb of a woman other than his wife with whom he has not had sexual relationship, thus making her pregnant, the resultant child belongs, in terms of lineage ( nasab ), to the owner of the sperm. The sperm of the man is considered the cause of producing this child, while no illegitimate sexual relationship has existed between the owner of the sperm and the woman who became pregnant by his sperm (6,21,40,53). 

### Social father

One of the most important issues discussed in AID is the relation of the baby born to an infertile man whose wife utilized donated sperm. Can the infertile man consider himself to be the father of the child and be entitled all paternal rights and obligations? 

### First viewpoint

#### Apparent link

A number of lawyers believe that the child does not have a real, genetic link to the infertile man ( the mother’s husband ). However according to the farash rule, the child is apparently connected to the mother’s husband and all paternal rights apply. 

Answer: The principle of farash is considered a legal principle on which the Holy Prophet ( pbuh ) of Islam and his immaculate family have relied. The subject of these traditions is the married woman who, while married and has sexual intercourse with her husband, becomes pregnant through intentional or unintentional sexual deviation. Her husband may raise the question that whether his wife’s pregnancy is out of his own sexual intercourse or is as a result of her illegitimate relationship with others. The statement of the immaculate Imam ( pbuh ): "al-walad li -lfarash wa li -lahir alhajar" ( the baby is from the marriage bed and an adulterer should be stoned ) deals with the issue that the child belongs to the husband and as a result its illegitimacy will be improbable. 

Use of the word farash in this principle is because the Holy Quran and other religious sources sometimes directly refer to couples or with such implicit words as "garment ( libas )" and "farash". "They are your garments and you are their garments" (54) "and on thrones ( of dignity ), raised high" (55). The couples are considered as "garments" that cover up each other’s faults and as "beds" on which they sleep ( alluding to intercourse ). 

In lexical texts there are two definitions for the word farash: the wife or anyone who plays this role as a slave-girl/kaniz (12) and couples (56). Jurists have also used this word with either of these two meanings: sexual intercourse ( waty ) and marriage with the possibility of intercourse (20,21,33,47,57,58); because a question has often posed by Muslim jurists that whether the accomplishment of farash depends upon the husband’s sexual intercourse with his own wife, or if the man and woman are married and the possibility of intercourse exists for them, the farash, although uncertain, has taken place. 

In any case, the principle of farash is a jurisprudence rule when there is a suspicion as to whether the child was born as a result of the husband’s intercourse or an illegitimate relationship (8,32,59). The implication is an external judgment about the legitimacy of the child and its association with the husband as well as apparent denial of the possibility of producing a child out of an illegitimate relationship (12). 

Therefore, if the possibility of sexual intercourse between the husband and his wife exists even though one is not certain of its implementation, the child will belong to the husband unless he repudiates the child by liʿan ( oath of imprecation ) (18). If the husband is certain that he has not transferred his sperm to the womb of his wife neither through sexual intercourse, nor further procedures, then according to farash principle the child cannot be attributed to him even the sexual relationship may have taken place (8). It is with regard to this aspect that in the legal Shiʿite literature, the possibility of the attribution of the child to the husband in normal situations is accepted as an assumption (60). However from the viewpoint of Sunni jurists the child is attributed to the husband even if he did not have sexual intercourse with his offender wife and did not transfer his sperm to her womb by any means (12). 

Therefore citing the rule of farash in AID is completely inappropriate because the assumption is that the husband is responsible for infertility and because of this he needs donated sperm. We do not doubt that whether the child belongs to the husband or the sperm donor, but we are certain that the child belongs to the sperm donor. 

If we are certain that the child has no biological relation to the infertile man, we cannot say that the child is apparently attributed to him. Clearly, acquiring an identification card under the name of his own family for a child that belongs to others is not to be taken as a justification for genetic attribution and establishing lineage (61). The husband’s agreement with inseminating sperm donated by a stranger into his wife’s egg would not indicate the child’s apparent link to him. 

### Second viewpoint

#### Lineage discontinuity

According to jurisprudence rules and Islamic law there is no lineage relation between the child and the husband ( infertile man ) of the mother. None of the rights and responsibilities that exist between a father and his own child are applicable because alimony ( nafaqa ), heritage ( werasat ), custody ( hizanat ) and guardianship ( welayat ) do not exist between them (24). The exception is marriage, which is forbidden if the child is a girl according to the Quranic verse: "Forbidden to you are your mothers… and your step-daughters who are in your guardianship ( born ) of your wives to whom you have gone in, but if you have not gone in to them there is no blame on you ( in marrying them )" (62). The husband cannot marry the daughter of his wife ( rabibah ) (24). For additional information on this subject, please refer to references (31,63,66). 

The reason for discontinuity of the lineage is that according to scriptural texts "… nor has He made those whom you assert to be your sons your real sons, these are the words of your mouths …" (67), adoption ( tabanni ) is not permissible and has no social and legal efficiency (5,68,71). Such children are only the children of their real, biological father. "Assert their relationship to their father, this is more equitable with Allah, but if you do not know their fathers, then they are your brethren in faith and your friends …" (72). 

### Mother

If a woman, due to infertility attributed to her husband uses donated sperm for having a child, will she be the mother of the child? To answer this question we have to reassess the discussion of illegal transfer of the husband’s sperm to an unknown woman. Earlier we have explained that if a woman, by tribadism ( lesbian sex ) transfers her husband’s sperm to another woman and the second woman becomes pregnant in this way, there is no doubt that the child will have lineage relation to the owner of sperm. The first woman who transferred her husband’s sperm to the other women is, therefore, not the mother of the child (47). But with this assumption, is the second woman who has become pregnant from this sperm and delivered a child considered to be the child’s mother? There are two possibilities offered in jurisprudence texts. 

### Discontinuity of lineage

A number of jurists maintain that for establishment of kinship and continuity of nasab there must be marriage ( nikah ) between the man and the woman or at least with the suspicion of the existence of a marriage relationship, they perform sexual intercourse and reproduction ( watye bi shobha ). However since none of these points are available in this case, the child is considered to be illegitimate ( Haramzadeh ) and does not have any lineage relation with the woman who has given birth (7,73). 

### Lineage continuity

In contrast, some jurists maintain that the woman who receives the sperm that results in the birth of a child is considered the mother of the child. Therefore all legal effects of maternal relationship will apply (7,51,52). 

The title of "offspring" ( walad ) applies to this baby, for it has developed in the womb of this woman (6,17,60). On the other hand, the only impediment to the accomplishment of a descendant relationship is sexual deviation, otherwise known as an illegitimate relationship ( adultery ). As the child has not been born from this relationship, thus there is no reason to reject the child’s attribution to its mother and not to adhere to the legal consequences (7,73). 

By taking into consideration both possibilities, we comment about the relative correlation between the child and his mother in the issue of AID. As the studies show, there are two viewpoints among Muslim lawyers. 

### First viewpoint

#### Separation between awareness and unawareness of the woman

According to this viewpoint, if the woman based on her ignorance of this rule or subject uses donated sperm, hence the resultant child is considered to be a dubious child ( walad al-shobha ) and joins her in respect to lineage. However if she has knowingly used donated sperm for fertilization, the child will be regarded as an illegitimate child ( Haramzadeh ) and have no connection with her in terms of lineage (53). 

Answer: Earlier we have explained that the relationship between the child and the woman or man who prepared the grounds for its existence, is a genetic relationship. The legislator has no role in its existence or nonexistence. However he may not recognize some of its outcomes. 

Therefore, the child born through AID with by either willing or unwilling contribution of the sperm donor, is attributed to the woman. Since the relation between the woman and the sperm donor is not illegal ( adultery ), none of the legal outcomes of kinship correlation between them are accepted. Therefore awareness ( ilm ) or unawareness ( jahl ) of the woman about AID, if conceivable, has no effect on the continuity of lineage of the child with her. 

### Second viewpoint

#### Continuity of lineage (nasab)

According to this viewpoint which is the most accurate in terms of previous explanations, the child born through AID is the real and actual child of the woman whose sex cell contributed to the creation of this child who developed in her womb and came to life in this world (5,52). 

## Discussion

Citing the rule of farash for proving the relationship of a child is only permissible when there is the possibility of attribution of child to the husband. It is not possible to prove the lineage ( nasab ) of a child born through AID by farash because we have no doubt that the child has been born without contribution of the husband’s sperm. 

An infertile man whose wife has a baby by donated sperm, has no kinship with the child. The sperm donor is the real father of a child who is born by contributing his sperm and all paternal rights and responsibilities are applicable between them. Awareness or unawareness of the sperm donor about his germ cell being the origin of producing of a human being has no effect on the attribution of the child to the sperm donor. The use of donated sperm of known or unknown origins does not affect the attribution of the child to the sperm donor. 

A woman whose egg has been fertilized by a stranger’s sperm is the real mother of the child. Paternal rights and responsibilities are applicable between them. A child born by AID is a legitimate child and entitled to all rights for legitimate children. 
